# Geniposide for treating atherosclerotic cardiovascular disease: a systematic review on its biological characteristics, pharmacology, pharmacokinetics, and toxicology

**DOI:** 10.1186/s13020-024-00981-3

**Published:** 2024-08-20

**Authors:** Dexiu Li, Xiaoya Li, Xiaonan Zhang, Jiye Chen, Zeping Wang, Zongliang Yu, Min Wu, Longtao Liu

**Affiliations:** 1 Xiyuan Hospital, China Academy of Chinese Medical Sciences, Beijing, China; 2 National Clinical Research Center for Chinese Medicine Cardiology, Beijing, China; 3 Beijing University of Chinese Medicine, Beijing, China; 4 Guang’an Men Hospital, China Academy of Chinese Medical Sciences, Beijing, China

**Keywords:** *Gardenia jasminoides Ellis*, Geniposide, Atherosclerotic cardiovascular disease, Pharmacology, Pharmacokinetics, Toxicology

## Abstract

In recent years, the prevalence and fatality rates of atherosclerotic cardiovascular disease have not only shown a consistent rise that cannot be ignored, but have also become a pressing social health problem that requires urgent attention. While interventional surgery and drug therapy offer significant therapeutic results, they often come with common side effects. Geniposide, an active component extracted from the Chinese medicine *Gardenia jasminoides Ellis*, shows promise in the management of cardiac conditions. This review comprehensively outlines the underlying pharmacological mechanisms by which geniposide exerts its effects on atherosclerosis. Geniposide exhibits a range of beneficial effects including alleviating inflammation, inhibiting the development of macrophage foam cells, improving lipid metabolism, and preventing platelet aggregation and thrombosis. It also demonstrates mitochondrial preservation, anti-apoptotic effects, and modulation of autophagy. Moreover, geniposide shows potential in improving oxidative stress and endoplasmic reticulum stress by maintaining the body’s antioxidant and oxidative balance. Additionally, this review comprehensively details the biological properties of geniposide, including methods of extraction and purification, as well as its pharmacokinetics and toxicological characteristics. It further discusses the clinical applications of related biopharmaceuticals, emphasizing the potential of geniposide in the prevention and treatment of atherosclerotic cardiovascular diseases. Furthermore, it highlights the limitations of current research, aiming to provide insights for future studies.

**Graphical Abstract:**

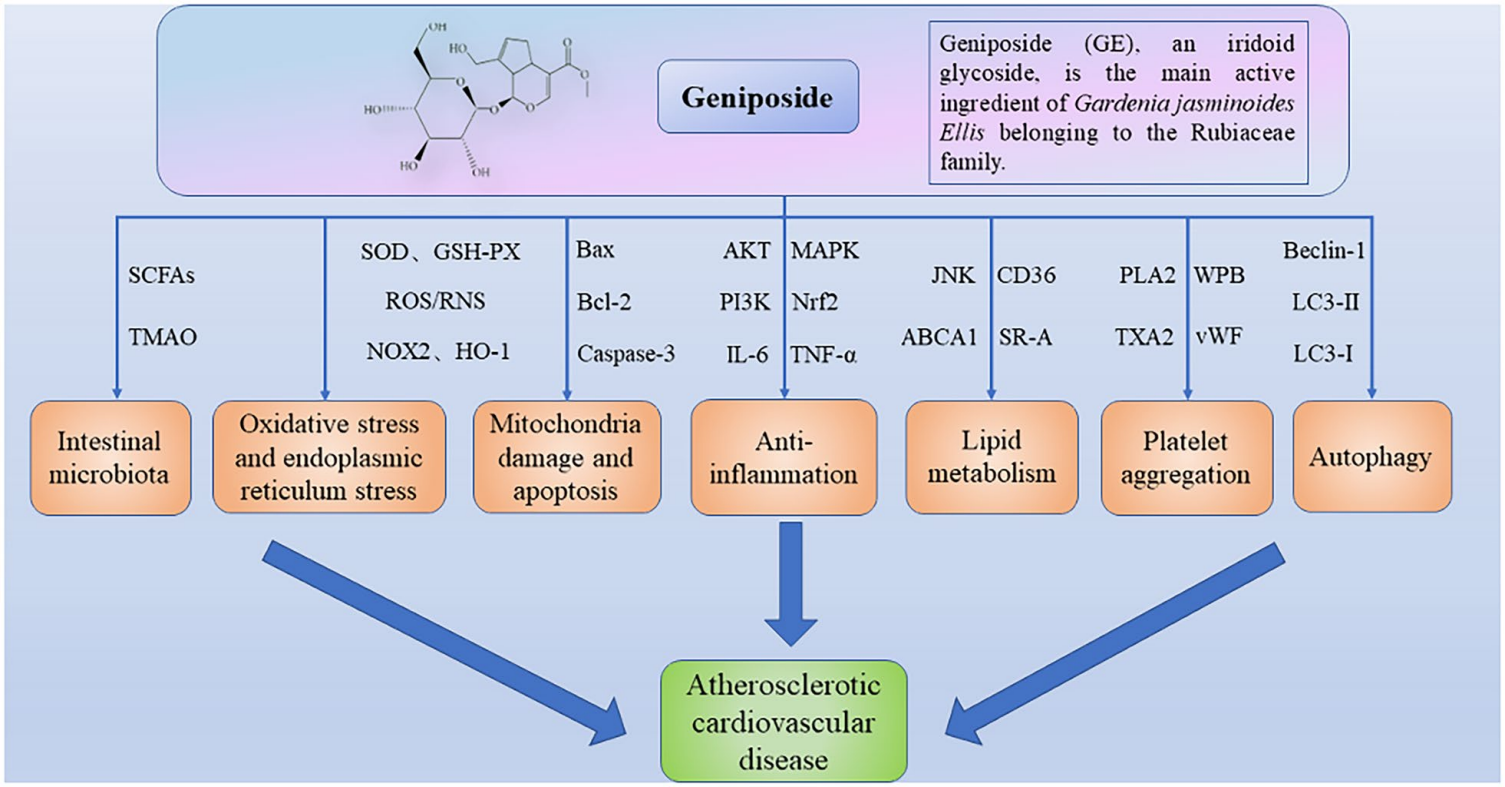

## Highlights

- Geniposide is an active iridoid glycoside compound extracted from the Chinese medicine *Gardenia jasminoides Ellis*.
- Geniposide can combat atherosclerosis through various mechanisms.
- Geniposide modulates immune cells and the reverse cholesterol transport, thereby limiting the formation of macrophage foam cells and improving lipid metabolism.
- The effects of geniposide on autophagy and apoptosis in VSMCs may be the emphasis of additional research.
- Whether geniposide can regulate the interaction between apoptosis and autophagy in CVD needs further investigation.
- Whether geniposide can target intestinal flora to inhibit atherosclerosis development needs further investigation.
- By selecting an appropriate route of administration and dosage regimen for geniposide, potential safety concerns can be effectively addressed.

## Introduction

Atherosclerosis is a progressive pathological process characterized by the accumulation of lipids and/or fibrous material in the intima of large- and medium-sized muscular arteries. Over time, the middle layer of the artery undergoes gradual deformation and calcification, resulting in the incrassation and induration of the artery wall and lumen stenosis. Once the vascular lumen is blocked, hypoxia and necrosis occur in the tissues and organs, leading to atherosclerotic cardiovascular diseases such as acute coronary syndromes [1]. Advancements in medical technology have introduced methods like interventional surgery and revascularization, which are widely applied in the treatment of acute cardiovascular events, significantly alleviating angina pectoris. However, these methods may not be suitable for all patients due to their associated high risks, heavy financial burden, and potential complications for the patients. Early and mid-stage patients with atherosclerosis often adopt drug therapy for prevention, which is convenient and economical but comes with significant side effects, such as strong drug resistance, rhabdomyolysis, and vascular restenosis, when taken long-term [2–5]. Natural compounds, on the other hand, can regulate multiple pathological processes of atherosclerosis through multiple targets and pathways, with fewer side effects. They can serve as an effective supplement for long-term management, helping to reduce the risk of disease recurrence and improve overall health [6].

**Fig. 1:**
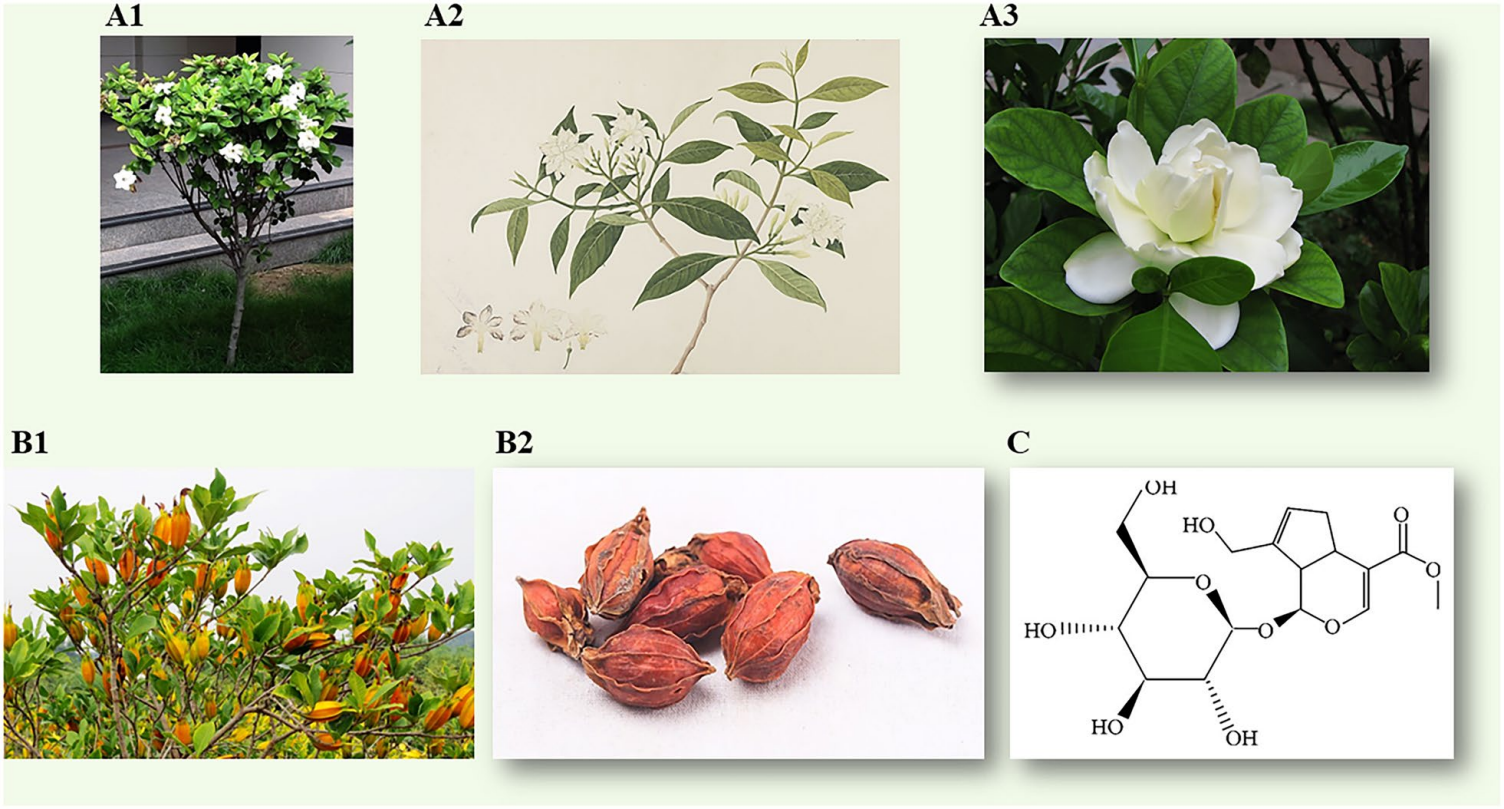
Whole plant and fruit of Gardenia jasminoides Ellis. **A1** and **A2** G. jasminoides plant; **A3** G. jasminoides flower. **B1** The ripe fruit; **B2** The desiccative fruit. **C** Geniposide

*Gardenia jasminoides Ellis* (G. jasminoides) is an evergreen shrub belonging to the Rubiaceae family, thriving in diverse regions worldwide. Officially licensed by the Ministry of Health of the People’s Republic of China, G. jasminoides recognized as the inaugural medicinal homologous foodstuff plant resource, holding significant botanical and medicinal status. The dehydrated ripe fruit of G. jasminoides, known as Zhizi, has been a staple in Asian folk medicine for millennia. Characterized by a bitter taste and cold nature, it acts on the heart, lung, and triple burner (San Jiao) meridians (Chinese Pharmacopoeia, 2020). It exhibits clinical efficacy, including the elimination of fire and detoxification, removal of pathogenic heat from the blood, drainage of dampness, and external application for detumescence and analgesia [7]. G. jasminoides can lower blood pressure and lipids, reduce blood sugar, protect the liver, and provide analgesic and sedative effects, improving sleep quality. Additionally, it possesses anti-inflammatory, antidepressant, antioxidant, antithrombotic, and anti-angiogenic pharmacological properties [8], all of which are closely related to its active constituents. G. jasminoides can be isolated into several chemical components, containing iridoid glycosides, triterpenoids, flavonoids, organic acids, pigments, and volatile compounds [8–10]. Among the aforementioned iridoid glycosides, geniposide stands out as the most plentiful iridoid glycoside in G. jasminoides and can be converted to genipin by β-D-glycosidase derived from intestinal bacteria [11]. The chemical structure is depicted in Fig. 1.

Recent research has shown that geniposide possesses pharmacological properties in combating inflammation, oxidative stress, apoptosis, and thrombosis [12]. It is extensively utilized as a natural medical remedy for various ailments, such as arthritis, diabetes, Alzheimer’s disease, and cardiovascular disorders [13–16]. Particularly, geniposide finds extensive application in treating cardiovascular conditions, including atherosclerosis, myocardial ischemia/reperfusion injury, myocardial fibrosis, and thrombosis within the cardiovascular system [17–21]. Despite these promising characteristics, there is a lack of systematic studies reviewing the effects of geniposide on atherosclerosis. Hence, this review aims to comprehensively summarize the biological properties, pharmacological mechanisms, toxicology, and pharmacokinetics of geniposide, thereby providing valuable insights for further research endeavors.

## Materials and methods

A comprehensive search of online academic databases, including PubMed, Web of Science, China National Knowledge Infrastructure (CNKI), and Wanfang Databases, was conducted using combinations keywords such as “geniposide”, “genipin”, “*Gardenia jasminoides Ellis*”, “Zhizi”, “atherosclerosis”, “cardiovascular diseases”, “pharmacology”, “pharmacokinetics”, and “toxicity”. Literature was initially included based on these keywords and gradually excluded if it did not align with the paper’s research theme after examining the titles, abstracts, and full texts. The final selection primarily covers studies from 2013 to 2023, with several seminal studies from earlier periods also incorporated.

### Biological characteristics of *Gardenia* jasminoides Ellis

#### Botanical characteristics

G. jasminoides is native to subtropical and tropical regions worldwide, notably prevalent in China, particularly in areas south of the Yangtze River. This shrub typically attains a height ranging from 60 to 180 cm, featuring gray bark and having glossy bottle-green leaves with prominent veins. The leaves are arranged oppositely, taking the form of elongated ovals measuring 4–10 cm in length, displaying a lustrous appearance. The flowers are fragrant, white, solitary, or paired, reaching up to 10 cm in diameter, and typically blossom during spring and summer months [22]. The fruiting body is oval, 1.5–3.5 cm in length and 1–1.5 cm in diameter, displaying a brownish-red hue and characterized by six wing-like longitudinal ribs. The fruit possesses a slight odor and a somewhat sour and bitter taste. The pericarp is thin, crisp, and slightly glossy. The seeds, densely covered with tiny verrucous projections, are crimson or reddish-yellow, oval, and clustered in groups (Chinese Pharmacopoeia, 2020). Details on the characteristics and fruit of G. jasminoides are shown in Fig. 1**.**

#### Extraction and quality control methods of geniposide

The major geniposide extraction methods encompass vapor fractionation as well as methods such as solvent, enzymatic, and ultrasonic- and microwave-assisted extraction [8, 23, 24]. Solvent extraction stands as the most prevalent method for geniposide extraction. Multiple hydrophilic chemical constituents, containing hydroxyl functional groups of geniposide, may be linked to its observed properties (Fig. 1). Ultrasonic and microwave extraction have the advantages of robust permeability, shortened processing time, and high yield, which are crucial for industrial production. The optimal yield of geniposide extracted from G. jasminoides via ultrasound-assisted extraction is achieved by utilizing water as the solvent, maintaining a solid–liquid ratio of 1:30, and performing the extraction at a temperature of 70 ℃ for 30 min. Impressively, under these conditions, the geniposide content escalates to a remarkable level of 40.31 ± 1.14 mg/g [24]. Geniposide and gardenia yellow have comparable solubility and are routinely extracted in combination, regardless of the solvent employed. The selective extraction method, in conjunction with mechanochemistry and solvent extraction, can enhance the yield of plant active components. The planetary ball mill operates at a speed of 200 rpm for a duration of 5 min, during which it mashes the powdered fruits of G. jasminoides with 30 wt% active carbon. The mixture is then subjected to water extraction (with a liquid–solid ratio of 10:1) at a temperature of 20 ℃ for 5 min, resulting in an 85% yield of the overall geniposide content [23]. Enzymatic extraction stands out for its simplicity, high efficiency, and environmental friendliness. The process relies on ultrasonic- and microwave-assisted pretreatment, combined with enzymatic hydrolysis and extraction (EHSE), offering an appealing alternative for the extraction and preparation of geniposide [25]. In recent years, researchers have widely studied combinations of multiple extraction methods [26]. The exploration of optimal combinations of extraction methods to maximize the yield of geniposide continues to an active area of research.

Geniposide (C_17_H_24_O_10_), an active component of G. jasminoides fruit, is a critical determinant for the quality control of Chinese Pharmacopoeia 2020. According to the national standard, in order to meet the criteria for acceptable quality, G. jasminoides should have a minimum geniposide concentration of 1.8%, as determined by HPLC (General Rule 0512) [27]. The wavelength of 238 nm is determined using octadecylsilane-bond silica gel as the stationary phase and a mobile phase consisting of acetonitrile–water (15:85). Geniposide content is quantified based on absorbance at 238 nm (Chinese Pharmacopoeia, 2020). Wu et al. collected 34 batches of G. jasminoides fructus samples from 10 provinces across China and meticulously analyzed the content of geniposide [28]. The study showed that the concentration of geniposide in the samples ranged from 3.6% to 4.1%, which fell within the limits specified by the Chinese Pharmacopoeia 2020. However, there is no significant variation in geniposide content across different production regions. As such, it is imperative to identify additional chemical markers to refine current quality control standards and differentiate G. jasminoides originating from diverse sources.

### The mechanism of geniposide in atherosclerotic cardiovascular disease

#### Inflammation

Inflammation performs an indispensable role in the pathogenicity mechanism of several cardiovascular diseases [16, 29–31]. Atherosclerosis therapy typically targets inflammation, for which cytokines are essential mediators and accompany atherogenesis at all stages [32]. Geniposide exerts anti-inflammatory effects on various diseases including arthritis [33], colitis [34], diabetes [35], depression [36], and Alzheimer’s disease [37]. Numerous studies have shown that geniposide can slow down the progression of coronary atherosclerotic cardiovascular disease through modulating the release of inflammatory cytokines [38].

The accommodation of inflammatory cytokines by geniposide has a correlation with the regulation of multiple inflammatory pathways. Geniposide can inhibit the release of inflammatory factors (NF-κB, TNF-α, and IL-1β) by activating Sirtuin (Sirt1) pathway, thereby attenuating obesity-related myocardial inflammation [39]. Shi et al. discovered that geniposide primarily exerts anti-inflammatory actions by suppressing the MAPK, AP-1, and NF-κB signaling pathways induced by LPS in macrophages. This subsequently reduces the overexpression of COX-2 and iNOS, and inhibits the release and expression of pro-inflammatory mediators and factors, including TNF-α, IL-6, NO, and PGE_2_ [40]. During atherosclerosis progression, phosphorylated activation of p38 MAPK enhances the exudation of pro-inflammatory cytokines and exacerbates vascular inflammation [41]. Liu et al. demonstrated that geniposide effectively attenuates the LPS-induced migration of HUVECs and inhibits U937 monocytes adhesion to the HUVECs. Geniposide reduces the phosphorylation levels of ERK1/2 and p38 MAPK, thereby impeding the decomposition of IkB-α and the activation of NF-κB, and ultimately suppressing the generation of IL-6 and IL-8 in LPS-induced HUVECs [42]. Geniposide can be assimilated in the gut and converted into genipin, which means that genipin is the main component of geniposide in the bloodstream [43]. Research investigates the inhibitory effects of genipin on LPS-induced inflammation in RAW264.7 murine macrophages through activation of the PI3K-JNK1/2-Nrf2 signaling cascade. Geniposide facilitates JNK1/2 and Nrf2 phosphorylation by activating the PI3K, resulting in increased heme oxygenase-1 (HO-1) expression and ultimately enhancing its anti-inflammatory efficacy [44]. TLR4 is capable of inducing the secretion of pro-inflammatory mediators [45]. Geniposide can downregulate TLR4 expression to attenuate inflammation response in mouse models induced by LPS, via inhibiting the NF-κB and the release of TNF-α, IL-1β, and IL-6 [46].

The Wnt1 signaling pathway can promote the proliferation and angiogenic function of cultured HUVECs [47], while the DKK1 signaling pathway can promote platelet-mediated inflammation and destabilize plaque in atherosclerosis [48]. Geniposide regulates inflammation and prevents atherosclerotic injury by increasing Wnt1, decreasing DKK1 expression in ox-LDL-stimulated HUVECs, and further inhibiting NF-κB and IL-12 expression. It can also reduce the serum total cholesterol (TC) and low-density lipoprotein cholesterol (LDL-C) levels, as well as the atherosclerotic lesion area, in ApoE^−/−^ mice with a high-fat diet (HFD) [49]. Numerous studies have verified the essential function of pyrin domain-containing protein 3 (NLRP3) inflammasome in atherogenesis following their activation [50]. Experimental results revealed that geniposide decreases the TC, triglyceride, and LDL-C levels, and increases the high-density lipoprotein cholesterol (HDL-C) levels in ApoE^−/−^ mice with HFD. It also shrinks the lesion area of aortic root plaque, the area of necrotic core in aortic plaque, and augment the area of collagen fiber. And these changes are related to the trigger of the AMPK/mTOR/Nrf2 pathway by geniposide, which subsequently weakens the activity of NLRP3 inflammasome and reduces the release of inflammatory factors and mediators [51]. Furthermore, geniposide exhibits a significant effect in enhancing myocardial ischemia/reperfusion injury (MI/RI) and infarct size, while concurrently limiting the release of IL-18, IL-1β, and caspase-1 in a mouse model. This occurs through the activation of the AMPK pathway, which regulates inflammation and pyroptosis mediated by ROS/TXNIP/NLRP3 pathways [52].

The inhibition of inflammation by geniposide may be associated with regulating the microRNA (miRNA) expression. MAPK phosphatase-1 (MKP-1) can inhibit inflammatory injury in vascular endothelium by deactivating the p38 MAPK phosphorylation [53]. Cheng et al. demonstrated that geniposide can block miRNA-101, leading to an enhanced expression of MKP-1 and subsequent attenuation of JNK, p38 MAPK, and ERK1/2 phosphorylation, along with decreasing the levels of IL-6 and TNF-α. Geniposide treatment has ameliorated the blood lipid levels and plaque areas in the atherosclerosis mouse model, improved the hepatic steatosis, and stabilized the plaques by increasing the collagen fibers within the plaques [54]. Similarly, geniposide can promote the miRNA-21/PTEN pathway in an ox-LDL-induced injury model using HUVECs. This leads to a decrease in pro-inflammatory cytokines levels and an increase in anti-inflammatory factor expression (IL‑10), effectively attenuating the inflammatory reaction and protecting against atherosclerosis damage [55].

Adhesion of monocytes, stimulated by adhesion factors, has a crucial effect on the progression of atherosclerosis. Genipin, in a dose- and time-dependent manner, enhances the production of peroxisome proliferator-activated receptor-γ (PPAR-γ), leading to varying degrees of reduction in the phosphorylation levels of PKC and AKT, inhibiting the expression of VCAM-1, and ultimately suppressing the TNF-α-induced adhesion of U937 monocytes to HUVECs [56]. Macrophages, differentiated from monocytes, exist in various stages of atherosclerosis and are involved in the generation of inflammatory mediators [57]. These macrophages can be polarized into two opposite functional phenotypes: M1 cells and M2 cells [58, 59]. Geniposide also regulates macrophage polarization through inhibiting the FOS/MAPK signaling pathway, resulting in a reduction in IL-1β associated with M1 phenotype and an elevation in IL-10 associated with M2 phenotype. This process improves the management of aortic intima hyperplasia, alleviates the plaque burden, and mitigates the severity of luminal stenosis, ultimately contributing to the stabilization of atherosclerotic plaques in a rabbit model [18]. The chemokine ligand 14 (CXCL14) promotes polarization of macrophages towards the M2 phenotype. Geniposide can enhance CXCL14 expression in the perivascular adipose tissue of ApoE^−/−^ mice, facilitating the transition of macrophages towards the M2 phenotype. This, in turn, leads to an augmentation in the content of collagen fibers within plaques, significantly enhancing their stability [60]. The activation of TLR4 can induce macrophage polarization towards the M1 phenotype [61], whereas geniposide exerts an inhibitory effect on the TLR4 signaling pathway. Consequently, it can be inferred that geniposide may regulate macrophage polarization by suppressing the TLR4 signaling pathway. Nevertheless, additional experiments are necessary to substantiate this hypothesis. Anomalous proliferation and migration of vascular smooth muscle cells (VSMCs) are the main cause of atherogenesis and restenosis [62]. Gao et al. have proved that geniposidic acid can protect ECs, promote ECs proliferation, inhibit VSMCs proliferation and migration, and prevent foam cell formation, thereby reversing vascular damage and plaque formation in the early stages of atherosclerosis [63]. HO-1, a cytoprotective enzyme and potent antioxidant, can suppress VSMCs proliferation and migration, and safeguard VSMCs against oxidative damage [64], and the benefits of upregulating HO-1 in atherosclerosis have been observed in various animal models [65]. A study demonstrated that genipin restrains ROS production and TNF-α-induced proliferation and migration of VSMCs, by increasing HO-1 expression and activity, mainly through preventing the phosphorylation levels of ERK1/2 and AKT pathways [66].

**Fig. 2:**
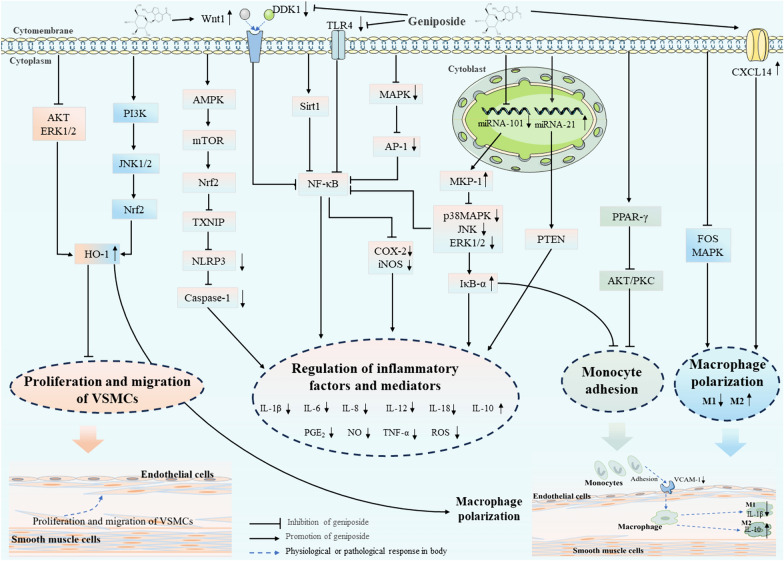
Pharmacological mechanisms of geniposide in atherosclerotic cardiovascular disease about anti-inflammation. Geniposide exerts anti-inflammatory effects through multiple pathways, such as modulating the release of inflammatory cytokines and mediators, obstructing the adhesion of monocytes to VECs, suppressing M1 macrophage polarization, promoting M2 macrophage polarization, and suppressing the proliferation and migration of VSMCs. *AKT* protein kinase B, *JNK* c-Jun N-terminal kinase, *MKP-1* mitogen-activated protein kinase (MAPK) phosphatase-1, *AP-1* activator protein-1, *TLR4* Toll-like receptor 4; *Sirt* sirtuin, *NF-κB* nuclear factor-kappa B; *DKK1* dickkopf-related protein-1, *PTEN* phosphatase and tensin homolog, *AMPK* adenosine monophosphate-activated protein kinase, *mTOR* mechanistic target of rapamycin, *Nrf2* nuclear factor erythroid 2-related factor, *TXNIP* thioredoxin-interacting protein, *NLRP3* pyrin domain containing protein 3, *PIK* phosphoinositide 3-kinase, *HO-1* heme oxygenase-1, *ERK* extracellular signal-regulated kinase, *PPAR-γ* peroxisome proliferator-activated receptor-γ, *PKC* protein kinase C, *CXCL14* Chemokine (C-X-C motif) ligand 14, *VCAM* vascular cell adhesion molecule; *ROS* reactive oxygen species

Table 1 summarizes the in vitro and in vivo experiments investigating the anti-atherosclerotic effects of geniposide, offering detailed experimental data and elucidating specific mechanism. In brief, geniposide modulates multiple signaling pathways, including MAPK, PI3K, ERK, JNK, AKT, TLR, mTOR, Nrf2, and NF-κB, and regulates the transcription of miRNA-101 and miRNA-21 to exert an anti-inflammatory effect **(**Fig. 2**)**. This regulation inhibits the release of pro-inflammatory mediators (NO and PGE_2_) and cytokines (IL-6, IL-8, IL-1β, and IL-12), promotes the release of IL-10 and chemokine (CXCL14). Furthermore, geniposide attenuates monocyte adhesion, VSMCs proliferation and migration, and promotes M2 polarization in macrophages during atherosclerosis progression.

**Table 1:** Experiments on the direct action targets and signal molecules of geniposide in atherosclerotic cardiovascular disease

Application	Experimental model	Medicine and dose	Group	Duration	Direct action targets of geniposide	Signaling molecules involved (GE group)	References
In vivo	High-fat-diet-induced male New Zealand rabbits	Geniposide (GE): 1.5 mg/kg/d	(1) Control: normal + NS; (2) Model: HFD + NS (the 8th–12th week); (3) GE: HFD + GE (the 8th–12th week)	12 weeks	FOS/MAPK↓	iNOS↓, IL-1β↓, Arg-1↑, IL-10↑, CD14↓, IL-1A↓, NR4A1↓	[18]
In vivo	High-fat-diet-induced obesity adult mice model	GE: 50 mg/kg, 0.2 ml	(1) Control (male C57/B6J mice) + NS; (2) Control + GE (the 22th–24th week); (3) HFD + normal saline (the 22th–24th week); (4) HFD + GE (the 22th–24th week)	24 weeks	Sirt1↑	AMPKα↑, GLP-1R↑, NF-κB↓, TNF-α↓, IL-1β↓	[39]
	Male C57/B6J mice with A*mpk* knockout		(1) Normal diet + siRNA (the 22th-24th week); (2) HFD + siRNA (the 22th-24th week); (3) HFD + GE + siRNA; (4) HFD + GE + siSirt1 (the 22th-24th week); (5) A*mpk* KO-HFD + GE + siRNA (the 22th–24th week); (6) A*mpk* KO-HFD + GE + siSirt1 (the 22th-24th week)				
In vitro	Neonatal rat cardiomyocytes (NRCMs)	GE: 50 μmol/L	(1) NRCMs + PA (500 μmol) + GE; (2) NRCMs + PA + GE + shAmpkα; (3) NRCMs + PA + GE + siSirt1; (4) NRCMs + PA + GE + Ex9-39; (5) NRCMs + PA + GE + siGlp-1r	24 h			
In vitro	LPS-induced murine macrophage RAW264.7 cells	GE: 40, 80, 160 μg/mL; 25, 50, 100, 200, 400 μg/mL	(1) Control; (2) LPS (50 ng/mL); (3) GE (25–400 μg/mL, 1 h prior) + LPS (50 ng/mL, 24 h); (4) GE (40–160 μg/mL, 1 h prior) + LPS (50 ng/mL, 4 or 12 or 20 h); (5) GE (80, 160 μg/mL, 1 h prior) + LPS/TNF-α/IL-1β (50 ng/mL, 12 h)	5–25 h	MAPK↓	NF-κB↓, AP-1↓, iNOS↓, COX-2↓, TNF-α↓, IL-6↓, NO↓, PGE_2_↓	[40]
In vitro	LPS-induced HUVECs	GE: 25, 50, 100 μg/mL	(1) Control; (2) LPS (100 ng/mL, 4 or 24 h); (3) GE (25–100 μg/mL, 24 h); (4) GE (25–100 μg/mL, 24 h prior) + LPS (100 ng/mL, 30 min, 1 or 4 or 8 h)	4–32 h	p38 MAPK↓, ERK1/2↓	NF-κB↓, IL-6↓, IL-8↓, IκB-α↑	[42]
In vitro	Murine macrophage RAW264.7 cells	Genipin: 10, 25, 50, 100, 200 μM	(1) Control; (2) Genipin (25–100 μM, 6 or 24 h); (3) Genipin (100 μM, 1 h, 3, 6, 9, 12, 24 h); (4) Genipin (10–200 μM, 24 h); (5) LPS (10 ng/mL, 24 h); (6) Genipin (100 μM, 30 min or 6 h) + LPS (10 ng/mL, 24 h)	1–30 h	PI3K↑	HO-1↑, JNK1/2↑, Nrf2↑	[44]
In vitro	LPS-induced murine macrophage RAW264.7 cells	GE: 5, 10, 15 mmol/L	(1) Control; (2) LPS (100 ng/mL); (3) GE (5–15 mmol/L, 24 h); (4) GE (15 mmol/L, 24 h prior) + LPS (100 ng/mL, 4 h)	24, 28 h	TLR4↓	NF-κB↓, IL-6↓, IL-1β↓, TNF-α↓	[46]
In vivo	High-fat-diet-induced male ApoE^−/−^ mice	GE: 100 mg/kg/d Baicalin (BAI): 200 μl/kg/d	(1) Control (C57BL/6 J) + NS; (2) HFD (C57BL/6 J) + NS; (3) HFD (ApoE^−/−^ mice) + BAI; (4) HFD (ApoE^−/−^ mice) + GE; (5) HFD (ApoE^−/−^ mice) + BAI + GE	12 weeks	Wnt1↑, DKK1↓	NF-κB↓, IL-12↓	[49]
In vitro	ox-LDL-induced HUVECs	GE/BAI: 5 mg/mL	(1) Control; (2) ox-LDL (50 mg/mL); (3) ox-LDL + BAI; (4) ox-LDL + GE; (5) ox-LDL + BAI + GE	48 h			
In vivo	High-fat-diet-induced ApoE^−/−^ mice	GE/Notoginsenoside R1 (NR): 50 mg/kg/d	(1) Control (male C57BL/6 J); (2) Model; (3) HFD + GE (the 13th-24th week); (3) HFD + NR (the 13th-24th week); (4) HFD + NR + GE (the 13th-24th week)	24 weeks	AMPK↑	mTOR↑, Nrf2↑, NLRP3↓, Bax↓, Bcl-2↑, caspase-3↓, caspase-1↓, IL-1β↓, IL-6↓, TNF-α↓, IL-18↓, ICAM-1↓, VCAM-1↓, NOX2↓, MDA↓, ROS↓, GSH↑, SOD↑	[51]
In vitro	H_2_O_2_-induced HUVECs	GE/NR: 12.5, 25, 50, 100, 200, 400 μM; H_2_O_2_: 0, 12.5, 25, 50, 100, 200, 400, 500 μM	(1) Control; (2) H_2_O_2_ (0–500 μM); (3) GE/NR (12.5–400 μM); (4) H_2_O_2_ (200 μM, 24 h) + GE/NR (25–100 μM, 24 h); (5) H_2_O_2_ (200 μM, 24 h) + GE + NR (50, 100 μM + 50, 100 μM, 24 h)	24, 48 h			
In vivo	MI/RI-induced male C57BL/6 J mice	GE: 10, 20 mg/kg/d	(1) Sham (similar operations without LAD ligature); (2) MI/RI + Vehicle; (3) MI/RI + GE (10 mg/kg/d); (4) MI/RI + GE (20 mg/kg/d)	24 h	AMPK↑	IL-1β↓, IL-18↓, caspase-1↓, ROS↓, TXNIP↓, NLRP3↓	[52]
In vitro	H/R-induced NRVMs	GE: 20, 40 μM	(1) Control; (2) H/R + Vehicle; (3) H/R + GE (20, 40 μM); (4) H/R + Compound C (20 μM); (5) H/R + AICAR (200 μM); (6) H/R + GE (40 μM) + Compound C/AICAR	4 h			
In vivo	High-fat-diet-induced ApoE^−/−^ mice	GE: 50 mg/kg/d; Simvastatin: 5 mg/kg/d	(1) Control (WT C57BL/6 J); (2) HFD; (3) HFD + Simvastatin; (4) HFD + GE	12 weeks	miR-101↓	MKP-1↑, p38 MAPK↓, JNK↓, IL-6↓, TNF-α↓	[54]
In vitro	RAW264.7 macrophage cell	GE: 2.5, 5, 10, 20, 40, 80 μM	(1) Control; (2) LPS (100 ng/mL, 1 h or 24 h); (3) (GE (2.5–80 μM, 24 h); (4) GE (2.5–80 μM, 4 h prior) + LPS (100 ng/mL, 24 h); (5) GE (10–80 μM, 4 h prior) + LPS (100 ng/mL, 1 h or 24 h);	1 h, 5, 24, 28 h			
In vitro	ox-LDL-induced HUVECs	GE: 5, 10, 20, 40, 80 μM	(1) Control; (2) ox-LDL (25, 50, 100 μg/mL, 24 h); (3) GE (1–80 μM, 30 min prior) + ox-LDL (50 μg/mL, 24 h); (4) GE (40 μM, 1 h prior) + ox-LDL (50 μg/mL, 24 h)	24–25 h	miR-21↑	PTEN↓, IL-6↓, IL-1β↓, TNF-α↓, IL-10↑, ROS↓, SOD↑, GSH-PX↑, CAT↑	[55]
In vitro	TNF-α-induced HUVECs	Genipin: 1, 5, 10, 50 μM	(1) Control; (2) TNF-α (10 ng/mL, 10 min, 30 min, 6 h, 24 h); (3) Genipin (1–50 μM, 30 min prior) + TNF-α (10 min); (4) Genipin (1–50 μM, 1 h prior) + TNF-α (6 h); (5) Genipin (1–50 μM, 24 h prior) + TNF-α (10 min or 30 min)	10 min-24.5 h	PPAR-γ↑	AKT↓, PKC↓, VCAM-1↓, ROS↓	[56]
	TNF-α-induced HUVECs + U937 monocytes		(1) Control; (2) TNF-α (10 ng/mL, 6 h); (3) Genipin (1–50 μM, 1 h prior) + TNF-α (6 h)	7 h			
In vivo	Western-diet- induced ApoE^−/−^ mice	GE: 50 mg/kg/d	(1) Control (wild-type mice); (2) WD + PBS; (3) WD + GE (12 weeks)	24 weeks	CXCL14↑	iNOS↓, IL-1β↓, IL-6↓, TNF-α↓, Arg1↑, IL-4↑, IL-10↑, TGF-β↑, IFN-γ↓	[60]
In vitro	TNF-α-induced VSMCs	Genipin: 25, 100 μM	(1) Control; (2) Genipin (25, 100 μM, 24 h); (3) TNF-α (100 ng/mL, 24 h); (4) Genipin (25, 100 μM, 1 h prior) + TNF-α (100 ng/mL, 24 h)	24, 25 h	ERK1/2↓, AKT↓ MAPK↓	ROS↓, HO-1↑, MMP-2↓, MMP-9↓, PAI-1↓, IL-8↓	[66]
In vivo	High-cholesterol diet-induced ApoE^−/−^ mice	GE: 100 mg/kg; BAI: 100 mg/kg	(1) C57BL/6 J control (standard mouse food); (2) ApoE^−/−^ control; (3) ApoE^−/−^ + BAI; (4) ApoE^−/−^ + GE	12 weeks	Foxp3↑	TGF-β1↑, IL-10↑	[68]
In vivo	High-cholesterol-diet-induced ApoE^−/−^ mice	GE: 100 mg/kg/d, 200 μL/kg/d; BAI: 100 mg/kg/d, 200 μL/kg/d	(1) ND (wild-type male C57BL/6 J mice, standard diet); (2) HCD; (3) HCD + BAI; (4) HCD + GE; (5) HCD + BAI + GE	12 weeks	CD11C↓, CD83↓	IL-12↓	[72]
In vitro	Bone marrow derived macrophages	GE: 0, 1, 10, 100 μM	(1) Control; (2) GE (0, 1, 10, 100 μM, 1 h) + ox-LDL (100 μg/mL, 24 h); (3) GE (1, 10, 100 μM, 1 h) + ox-LDL (100 μg/mL, 30 min);	1.5 h, 25 h	p38 MAPK↓, ERK↓, JNK↓	IL-6↓, TNF-α↓, MCP-1↓, iNOS↓, NO↓, CD36↓, p65↓, NF-κB↓	[74]
In vivo	High-fat-diet-induced ApoE^−/−^ mice	GE: 50, 100 mg/kg/d	(1) HFD + NS (final 4 weeks); (2) HFD + GE (50 mg/kg/d, final 4 weeks); (3) HFD + GE (100 mg/kg/d, final 4 weeks)	16 weeks	p38 MAPK↓, AKT↓	SR-A↓, SR-B1↑, ABCA1↑, CD68↓	[75]
In vitro	LPA-induced Raw264.7 macrophages	GE: 50, 100, 200 μg/mL	(1) Control; (2) LPA (200 μM); (3) LPA + GE (50, 100, 200 μM)	24 h			
In vivo	High-cholesterol-diet-induced ApoE^−/−^ mice	GE: 50 mg/kg/d	(1) C57BL/6 J mice + NCD (normal chow diet); (2) C57BL/6 J mice + NCD + GE; (3) C57BL/6 J mice + HCD; (4) C57BL/6 J mice + HCD + GE; (5) ApoE^−/−^ mice + HCD (13 weeks); (6) ApoE^−/−^ mice + HCD + GE (13 weeks)	4, 13 weeks	FXR↓	I-BABP↓, ASBT↓, SHP↓, HNF-4α↑, LRH-1↑	[79]
In vitro	Human HepG2 cells and Caco2 cells	GE: 100 μM; GW4064 (an FXR agonist)	(1) HepG2 (12, 24 h); (2) HepG2 + GE (12, 24 h); (3) HepG2 + GE + GW4064 (12, 24 h) (4) Caco2 (12, 24 h); (5) Caco2 + GE (12, 24 h); (6) Caco2 + GE + GW4064 (12, 24 h)	12, 24 h			
In vivo	Male Sprague–Dawley (SD) rats	GE: 100 mg/kg/d	(1) Sham group + NS; (2) I/R group + NS (30 min NS, 30 min ischemia, 2 h reperfusion); (3) I/R + GE group (30 min NS, 30 min ischemia, 2 h reperfusion)	3 h	AKT↑	ATG5↓, ATG7↓, Beclin-1↓, LC3-II/LC3-I↓, p62↑, Bax↓, Bcl-2↑, mTOR↓	[114]
In vitro	H9c2 cells	GE: 40 μM; RAPA: 100 nM	(1) Control; (2) Control + GE; (3) Control + RAPA; (4) H/R group (4 h reoxygenation + 12 h hypoxia); (5) H/R + GE (pretreated with GE for 30 min); (6) H/R + RAPA (pretreated with RAPA for 30 min); (7) H/R + GP + RAPA (pretreated with GP and RAPA for 30 min)	16.5 h			
In vivo	High-fat-diet-induced ApoE^−/−^ mice	GE: 50 mg/kg/d, RAPA: 2 mg/kg, twice a week, Simvastatin (SV): 5 mg/kg/d	(1) Control (male wild-type C57BL/6 mice) + NCD + NS; (2) HFD; (3) HFD + GE; (4) HFD + RAPA; (5) HFD + SV	24 weeks	mTOR↓	TREM2↓, Beclin-1↑, LC3-II/LC3-I↑, p62↓	[116]

↓ downregulation or inhibition, ↑ upregulation or activation, *NS* normal saline, *PA* palmitic acid, *iNOS* inducible nitric oxide synthase, *IL-S* Interleukins, *Arg-1* arginase-1, *NR4A1* nuclear receptor subfamily 4 group A member 1, *Sirt1* Sirtuin1, *GLP-1R* glucagon-like peptide-1 receptor, *AMPK* adenosine monophosphate-activated protein kinase, *NF-κB* nuclear factor-kappa B, *TNF-α* tumor necrosis factor α, *MAPK* mitogen-activated protein kinase, *AP-1* activator protein-1, *COX-2* cyclooxygenase, *NO* nitric oxide, *PGE*_*2*_ prostaglandin E2, *HUVECs* human umbilical vein endothelial cells, *ERK1/2* extracellular signal-regulated kinase, *HO-1* heme oxygenase-1, *PI3K* phosphoinositide 3-kinase, *JNK1/2* c-Jun N-terminal kinase, *Nrf2* nuclear factor erythroid 2-related factor, *TLR* Toll-like receptor, *DKK1* dickkopf-related protein-1, *mTOR* mechanistic target of rapamycin, *NLRP3* pyrin domain containing protein 3, *NRVMs* neonatal rat ventricular myocytes, *LAD* left anterior descending, *Bax* Bcl-2 associated protein X, *ROS* reactive oxygen species, *TXNIP* thioredoxin-interacting protein, *MKP-1* mitogen-activated protein kinase (MAPK) phosphatase-1, *PTEN* phosphatase and tensin homolog, *SOD* superoxide dismutase, *GSH-PX* glutathione peroxidase, *CAT* catalase, *PPAR-γ* peroxisome proliferator-activated receptor-γ, *AKT* protein kinase B, *PKC* protein kinase C, *VCAM-1* vascular cell adhesion molecule-1, *MMPs* matrix metalloproteases, *PAI-1* Plasminogen activator inhibitor-1, *VSMC* vascular smooth muscle cell, *CXCL14* Chemokine (C-X-C motif) ligand 14, *FXR* farnesoid X receptor, *ASBT* apical sodium-dependent bile acid transporter, *SR-A* scavenger receptor A, *SR-B* scavenger receptor B, *ABCA1* adenosine triphosphate-binding cassette transporter A1, *TREM2*, trigger receptor expressed on myeloid cells 2, *CD* cluster of differentiation 36, *FOXP3* Forkhead box protein P3, *ATG* autophagy protein

#### Lipid metabolism

Disorder in the metabolism of lipids represents an event in atherosclerosis, with elevated levels of TC and LDL-C in plasma serving as essential risk factors for its development [67]. Geniposide significantly reduces serum TC and LDL-C levels and alleviates atherosclerotic lesions in HFD mice [49]. Analogously, geniposide notably enhances Foxp3 expression and stimulates the proliferation of Treg cells, strikingly elevating the release of cytokines (TGF-β1 and IL-10) while decreasing the plaque areas. And it also ameliorates immunoregulation and lipid regulation [68]. Dendritic cells (DCs), present in atherosclerotic plaques and vascular adventitia [69], can phagocytose lipids, transform into foam cells [70], and promote the recruitment of monocytes and macrophages in atherosclerotic lesions [71]. Liu et al. verified that geniposide diminishes the number of DCs, decreases plasma lipid levels, and downregulates the expression of CD11C and CD83. These effects hinder DCs maturation and their infiltration into atherosclerotic plaques, ultimately inducing a regression of atherosclerotic lesions [72]. Macrophages can recognize and internalize oxidized lipids, and then turn into foam cells [73]. Geniposide alleviates the levels of cholesterol and LPL, suppresses ox-LDL-induced macrophage foam cell formation, and attenuates NO production, iNOS activity, and CD36 expression by preventing the activation of NF-κB and MAPKs signaling pathways [74]. Shen et al. demonstrated that geniposide observably reduces the concentrations of LDL-C, TG, and serum TC, while regulating the ingestion and transportation of cholesterol by improving expression of efflux-related proteins and lipoproteins. Furthermore, by upregulating SR-B1 and ABCA1, while downregulating SR-A, geniposide modulates the equilibrium of diverse lipid transporters. This modulation is achieved through the suppression of the AKT and p38MAPK signaling pathways, ultimately impeding the formation and accumulation of foam cell macrophage in lesion plaque [75].

**Fig. 3:**
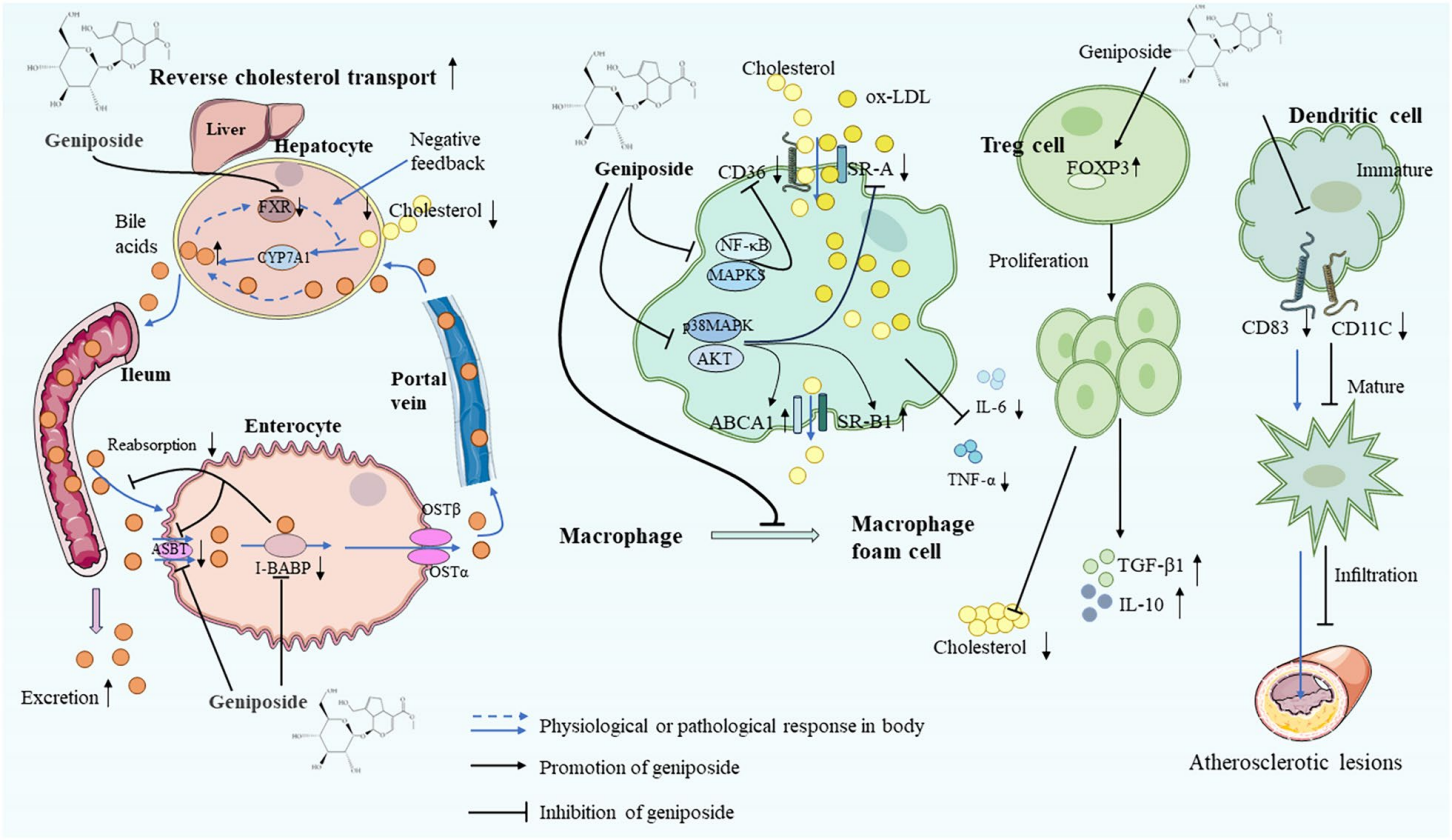
Pharmacological mechanisms of geniposide in regulating lipid metabolism. Geniposide modulates immune cells and reverse transport of cholesterol, and inhibits the formation of macrophage foam cells, thus improving lipid metabolism. *FXR* farnesoid X receptor, *CYP7A1* Cholesterol 7 alpha-hydroxylase, *ASBT* apical sodium-dependent bile acid transporter, *I-BABP* Ileal-bile acid-binding protein, *OST* organic solute transporte, *NF-κB* nuclear factor-kappa B, *p38MAPK* mitogen-activated protein kinase, *AKT* protein kinase B, *SR-A/B* scavenger receptor A/B, *ABCA1* adenosine triphosphate-binding cassette transporter A1, *CD* cluster of differentiation 36, *FOXP3* Forkhead box protein P3, *TGF-β1* transforming growth factor-β1

Reverse cholesterol transport plays an essential element in blocking the process of foam cell development [76]. Plasma cholesterol can be reversely conveyed to the liver and metabolized into bile acids [77, 78]. Geniposide inhibits Farnesoid X receptor (FXR) activity and reduces I-BABP expression, leading to the regulation of the enterohepatic circulation of bile acids. This mechanism involves blocking the negative feedback loop that controls bile acids levels in liver and their reabsorption in the ileum, thereby facilitating hepatic bile acid synthesis and excretion. Consequently, it accelerates the reverse cholesterol transport and catabolism, resulting in decreased blood cholesterol levels and improved atherosclerosis [79]. In summary, these results suggest that geniposide modulates immune cells and reverse cholesterol transport, inhibiting macrophages foam cells development and improving lipid metabolism. And its regulatory effects on lipid metabolism are illustrated in Fig. 3.

#### Platelet aggregation

Recent studies have consistently underscored the pivotal role of platelets in promoting thrombus formation subsequent to atherosclerotic plaque rupture [80]. Atherosclerotic plaque disruption can trigger a cascade of events, including local platelet activation, adhesion, aggregation, and the activation of the coagulation cascade, which ultimately culminate in the onset of atherothrombotic events [81]. An experimental investigation has revealed that geniposide exerts a multifaceted influence on thrombosis by not only reducing the dry and wet weight of thrombotic masses, but also diminishing whole blood viscosity, inhibiting endothelin (ET) release, and decreasing thromboxane B2 (TXB2) content, while simultaneously augmenting the levels of prostaglandin F1α (PGF1α) [82].

In situations, where the vascular endothelium is dysfunctional, the bioavailability of NO is reduced and levels of vWF are elevated, both of which result in platelet aggregation [83]. Exocytosis of endotheliocyte particles is one of the earliest reactions to vascular abnormalities, significantly impacting both inflammation and thrombosis [84, 85]. In this regard, geniposide emerges as a potent modulator, inhibiting thrombin-induced Weibel-Palade body (WPB) exocytosis in HUVECs by stimulating eNOS and NO production. This, in turn, leads to the mitigated release of vWF and translocation of P-selectin, eventually decreasing platelet adhesion and aggregation and suppressing thrombosis [86]. Zhang et al. have elucidated that geniposide shortens the length of the tail thrombus post-carrageenan injection and inhibits both arterial and venous thrombosis in rats by suppressing platelet aggregation mediated by collagen and thrombin [87].

**Fig. 4:**
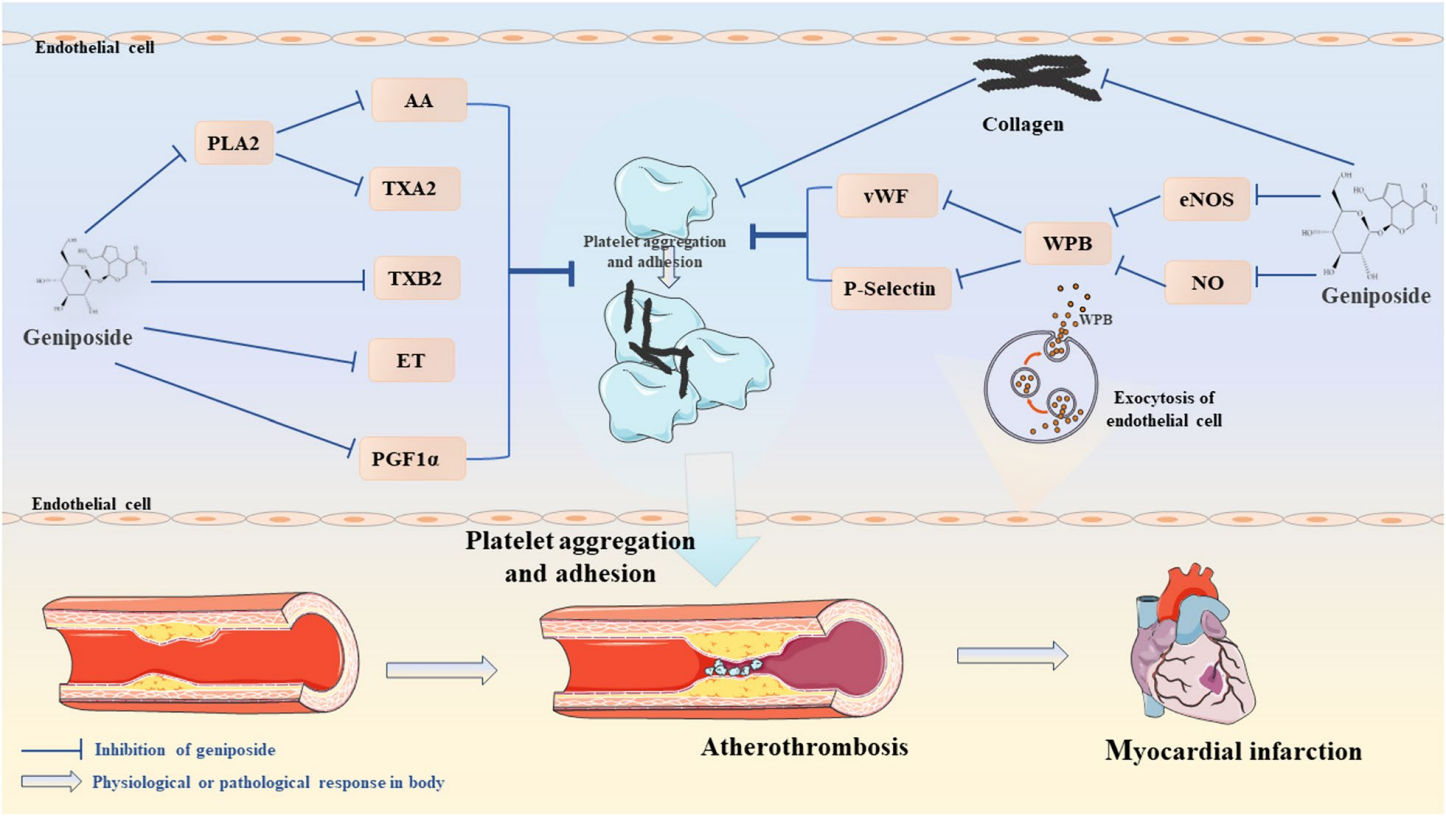
Pharmacological mechanisms of geniposide inhibiting platelet aggregation in atherosclerotic cardiovascular disease. Geniposide exhibits a powerful inhibitory function on collagen-induced platelet aggregation. *eNOS* endothelial nitric oxide synthase, *NO* nitric oxide, *WPB* Weibel-Palade bodies, *vWF* von Willebrand factor, *PLA2* phospholipase A2, *AA* arachidonic Acid, *TXA2/B2* thromboxane A2, *ET* endothelin, *PGF1α* prostaglandin F1α

Geniposide demonstrates a robust inhibitory effect on collagen-induced platelet aggregation. This phenomenon is potentially associated with the regulation of the release and metabolism of arachidonic acid (AA), a crucial mediator in platelet activation [88]. Further conjecture suggests that geniposide inhibits phospholipase A2 (PLA2) activity, suppresses AA release, and prevents thromboxane A2 (TXA2) generation, thereby inhibiting ADP-induced platelet aggregation [89]. Current evidence verifies that the inhibition of TNF-α, TLR4, and NLRP3 exerts an influence on platelet activation and aggregation. It is hypothesized that geniposide may attenuate the expression of inflammatory mediators, thereby alleviating platelet aggregation and ultimately reducing the risk of atherothrombosis [90–92]. In essence, geniposide inhibits platelet aggregation and thrombosis by regulating vWF, PLA2, and TXA2 pathways. Additionally, it likely diminishes the secretion of inflammatory mediators, further enhancing its protective role against thrombotic events (Fig. 4). These findings underscore the therapeutic potential of geniposide in the management of cardiovascular disorders associated with platelet hyperactivity.

### Mitochondria damage and apoptosis

Mitochondria are highly abundant in cardiomyocytes, utilizing oxygen for energy metabolism, and participating in pathophysiological responses throughout ischemia/reperfusion (I/R) injury. Reperfusion generates numerous oxidative stress products (ROS/RNS), inducing membrane depolarization, calcium overload, mitochondrial oxidative stress, and pro-apoptotic proteins secretion. Consequently, this cascade leads to cardiomyocyte apoptosis and death [93–95].

Accumulating evidence has revealed that mitochondria-dependent pathways are crucial in I/R-induced apoptosis of cardiomyocytes [96, 97]. Jing et al. showed that geniposide suppresses hypoxia/reoxygenation (H/R)-induced cardiomyocyte apoptosis by restoring mitochondrial function. This effect is associated with the GLP-1R-mediated activation of the PI3K/AKT signaling pathway, resulting in modulation of the Bax/Bcl-2/mitochondria apoptotic pathways. Specifically, the underlying mechanisms are that geniposide ameliorates mitochondrial dysfunction by diminishing the concentration of MDA and ROS, while raising the activity of antioxidant enzyme (T-SOD) in H9c2 cells. Furthermore, it downregulates the expression of Bax, upregulates the expression of Bcl-2, and inhibits caspase-3 activation, thereby preventing mitochondrial membrane depolarization and mitigating mitochondrial calcium overload [98]. Hou et al. verified that geniposide modulates the AMPK/Sirt1/FOXO1 signaling pathway to improve energy metabolism and regulates the p38/Bcl-2/Bax pathway to mitigate cardiomyocyte apoptosis induced by spontaneous hypertension [20]. Additionally, geniposide impedes the activation of AMPKα signaling [99, 100], reduces the Bax/Bcl-2 ratio, and inhibits caspase-3 cleavage [39], providing further substantiation for the impact of geniposide on cardiomyocyte apoptosis.

Vascular endothelial cells serve as the primary barrier in maintaining blood vessels integrity, and their apoptosis can expedite the progression of atherosclerosis [101]. Cardiovascular risk factors, such as oxidative stress, hyperlipidemia, and elevated lipopolysaccharide and homocysteine levels, can promote endothelial cell apoptosis and induce CVD [102–105]. Geniposide activates the AMPK/mTOR/Nrf2 pathway, leading to the inhibition of NADPH oxidase 2 (NOX2) and its subunit p22phox overexpression. This activation also reduces levels of MDA and ROS while increasing GSH levels and SOD activity. Additionally, geniposide downregulates the expression of Bax and caspase-3, while upregulating the expression of Bcl-2 protein, thus ameliorating the percentage of apoptosis in HUVECs [51].

In conclusion, geniposide exhibits a protective role against atherosclerosis by preserving mitochondria integrity and attenuating apoptosis. This mechanism is mediated through the regulation of the AMPK, Nrf2, and PI3K pathways, as well as the modulation of the Bax/Bcl-2/caspase-3 axis.

#### Autophagy

Autophagy, a ubiquitous lysosome-mediated degradation mechanism for protein turnover in cells, dismantles dysfunctional organelles and plays a crucial role in maintaining cellular homeostasis [106, 107]. Moderate autophagy supports cell self-repair and survival under stressful conditions [108]. Research has shown that the regulation of autophagy significantly affects vascular wall function and the occurrence and progression of CVD, including atherosclerosis [109]. Both excessive and impaired autophagy activity can impact atherosclerosis [110, 111]. Therefore, fine-tuning autophagic balance represents an effective therapeutic target for preserving cardiac and vascular homeostasis and function. Ample evidence indicates that excessive autophagy activation caused by myocardial I/R can induce cellular injury [112, 113]. Luo et al. used in vitro and in vivo models to investigate the protective action of geniposide on autophagy in cardiomyocytes subjected to I/R injury. Their results indicated that geniposide exerted suppressive effects on autophagy, myocardial injury, and infarct size, by activating the AKT signaling pathway and inhibiting the mTOR signaling pathway. In this experimental setup, geniposide hindered the secretion of autophagy-associated proteins (Beclin-1, ATG7, and ATG5), elevated the release of p62, reduced the LC3-II/LC3-I ratio, and prevented the formation and accumulation of autophagosomes in the I/R myocardium [114].

Impaired macrophage autophagy during atherosclerosis development suppresses the clearance of post-apoptotic macrophages and accelerates plaque necrosis [115]. Accordingly, promoting macrophage autophagy has the potential to enhance the stability of atherosclerotic plaques. Xu et al. confirmed that geniposide can inhibit plaque formation, impede atherosclerosis development, and promote extensive collagen deposition by elevating macrophage autophagy levels through the restraint of the mTOR and TREM2 signaling pathways in RAW264.7 macrophages [116]. In microvascular endothelial cells, geniposide enhances autophagy by upregulating Sirt3 to counteract H/R-induced EC injury [117].

In summary, geniposide plays a protective role in cardiovascular health by regulating autophagy through the AKT, mTOR, TREM2, and Sirt3 pathways, as well as through autophagy-related proteins. Geniposide can ameliorate apoptosis in osteoblasts and kidney podocytes by activating autophagy [118, 119]. Currently, no studies have confirmed whether geniposide can regulate apoptosis by activating autophagy or modulate the crosstalk between apoptosis and autophagy in the cardiovascular system, which is likely to be the focus of additional research on the cardiovascular protective mechanism of geniposide.

### Oxidative stress and endoplasmic reticulum stress

The increase in ROS production in vessel walls is correlated with dyslipidemia, diabetes, hypertension, smoking, and other risk factors for atherosclerotic cardiovascular disease [120]. Excessive ROS-induced oxidative stress is a fundamental and overarching mechanism underlying atherosclerosis [121]. Oxidative stress represents a prominent pathological manifestation of endoplasmic reticulum stress (ERS) [122, 123]. ERS occurs when unfolded or misfolded proteins accumulate in organelles and can be triggered by multiple pathological conditions [124]. ERS regulates endothelial dysfunction by interfering with the eNOS signaling pathway, while prolonged ERS triggers apoptosis and inflammatory pathways in macrophages and endothelial cells [125], thereby contributing to atherosclerotic plaque progression [126].

**Fig. 5:**
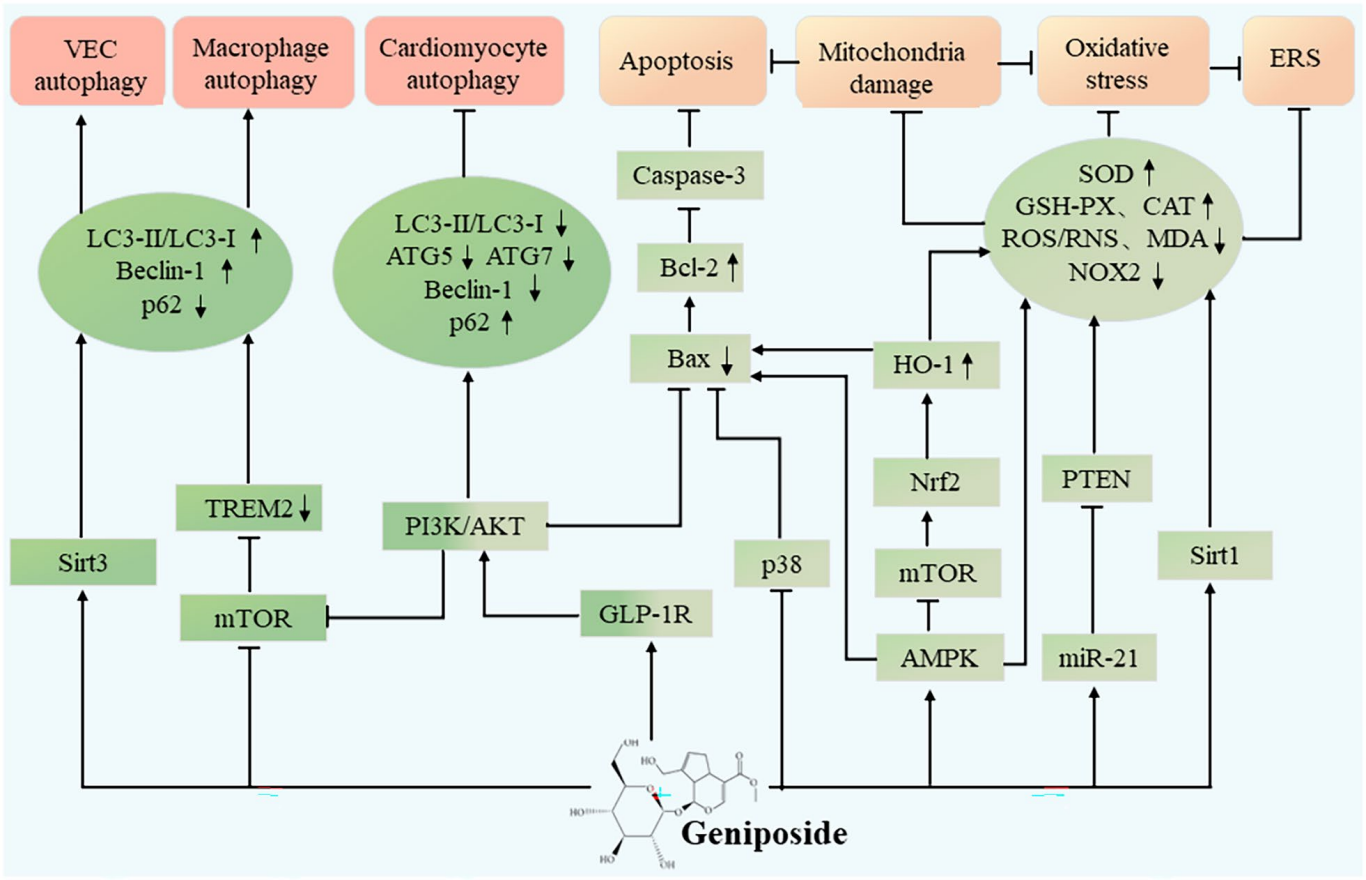
Geniposide possesses the ability to modulate oxidative stress products, thereby alleviating mitochondrial damage, oxidative stress, and ERS. Furthermore, it can regulate the apoptotic processes in cardiomyocytes, macrophages, and vascular endothelial cells. *Sirt* sirtuin, *mTOR* mechanistic target of rapamycin, *TREM2* trigger receptor expressed on myeloid cells 2, *p62* substrate ubiquitin binding protein of autophagy, *AKT* protein kinase B, *ATG* autophagy protein, *PI3K* phosphoinositide 3-kinase, *GLP-1R* glucagon-like peptide-1 receptor, *Bax*, Bcl-2 associated protein X, *AMPK* adenosine monophosphate-activated protein kinase, *Nrf2* nuclear factor erythroid 2-related factor, *HO-1* heme oxygenase-1, *PTEN* phosphatase, and tensin homolog, *SOD* superoxide dismutase, *GSH-PX* glutathione peroxidase, *CAT* catalase, *ROS* reactive oxygen species, *RNS* reactive nitrogen species, *MDA* malondialdehyde, *NOX2* nicotinamide adenine dinucleotide phosphate oxidase, *ERS* endoplasmic reticulum stress, *VEC* vascular endothelial cell

Studies have shown that geniposide inhibits oxidative stress and ERS in certain diseases [127–130]. Geniposide counteracts isoproterenol-induced cardiac fibrosis by activating the Sirt1 signaling pathway and blocking Smad3 acetylation, oxidative stress, and ERS [19]. Wang et al. demonstrated that the pretreatment with geniposide activates the Nrf2/HO-1 pathways, limiting oxidative stress and significantly alleviating MI/RI injury, as well as ameliorating myocardial systolic and diastolic function. Geniposide also suppresses ROS-mediated oxidative damage, downregulates MDA levels, strengthens the activities of antioxidants (GPx, SOD, and CAT), and maintains the body’s antioxidant-oxidative balance [21]. Additionally, geniposide mitigates the NOX2 expression to inhibit oxidative stress and promotes the antioxidant defense system through activating miRNA‑21/PTEN pathways [55]. The pharmacological mechanisms of geniposide in regulating oxidative stress, ERS, autophagy, mitochondria damage, and apoptosis in atherosclerotic cardiovascular disease are detailed in Fig. 5.

### Intestinal microbiota

Under physiological conditions, the intestinal microbiota can modulate bodily functions by releasing a diverse array of metabolites. Accumulating evidence reveals that the gut microbiota and its metabolites mediate protective effects against cardiovascular disease, including heart failure, atherosclerosis, and hypertension [131, 132], through a phenomenon commonly referred to as the “heart-gut axis” [133]. Nevertheless, an imbalance in the gut flora disrupts the production of valuable substances like short-chain fatty acids (SCFAs), compromises the integrity of the gut barrier, and leads to the release of numerous toxic substances, including LPS, phenylacetylglutamine, and trimethylamine-N-oxide (TMAO), into the bloodstream. Eventually, this dysbiosis exacerbates CVD progression [134].

TMAO can impede the hepatic transport of peripheral cholesterol, enhance platelet reactivity, promote thrombosis, facilitate the release of IL-6, TNF-α, and ICAM-1 [135], and activate the ROS-TXNIP-NLRP3 inflammasome [136]. As a result, it hampers endothelial cell function and exacerbates atherosclerosis through a process known as “intermingled phlegm and blood stasis”, which serves as a pathological basis for vascular obstruction [137]. On the other hand, SCFAs can improve lipid and glucose metabolism, activate the AMPK pathway [138], attenuate NF-κB activity, diminish pro-inflammatory cytokines production [139], and alleviate hypertension-induced pathological myocardial remodeling [140].

Traditional Chinese medicines have the potential to modulate the composition and metabolism of the intestinal microbiota, thus offering therapeutic benefits for digestive diseases, CVD, as well as nervous and respiratory disorders [141, 142]. Furthermore, through gut microbiota remodeling and downregulation of the enterohepatic FXR-FGF15 axis in atherosclerosis mice, TCM can inhibit TMAO synthesis [143]. Experimental studies have verified that geniposide counteracts inflammation by regulating intestinal bacteria and improving the gut barrier function [144, 145]. However, it is yet to be established whether geniposide can specifically target intestinal flora to impede AS development. Hence, geniposide shows promise as an emerging therapeutic approach for atherosclerosis and cardiovascular risk events by modulating pertinent factors within the gut flora, simultaneously downregulating the TMAO levels, and upregulating the SCFAs levels.

### Pharmacokinetics and toxicology of geniposide

#### Pharmacokinetics

Orally administered geniposide can undergo hydrolysis to genipin by β-D-glycosidase, an intestinal bacterial enzyme [11], and is primarily absorbed in the duodenum and jejunum via passive diffusion [146]. The bioavailability of geniposide may be influenced by its metabolism mediated by intestinal flora [147]. Wang et al. used gradient HPLC to investigate the bioavailability and tissue distribution of geniposide (100 mg/kg) after oral administration in rats. The maximum plasma concentration (C_max_) of geniposide was observed at 30 min (T_max_) after oral administration, with almost complete elimination from plasma within 12 h. Nevertheless, the absolute bioavailability of geniposide after oral administration was found to be only 9.67%. Regarding the tissue distribution of orally administered geniposide, C_max_ was detected in the spleen and liver at 0.5 h, in the plasma at 1 h, as well as in the brain and kidney at 2 h after administration. Tissue content analyses revealed that kidney had the highest accumulation, followed by spleen, liver, heart, lung, and brain, indicating a potential toxic accumulation in both liver and kidney [148]. Following oral ingestion of geniposide in male mice, a diverse array of metabolites was produced and widely distributed in the plasma, urine, liver, and kidney, with the most significant concentration in the urine [149]. Furthermore, sex-dependent differences were identified in geniposide metabolism; specifically, male rats exhibited enhanced absorption but reduced distribution and elimination compared to female rats [150].

The influence of dosage forms and administration methods on the pharmacokinetics of geniposide has been confirmed by studies. Lu et al. monitored the pharmacokinetic characteristics of geniposide administered intragastrically (i.g.), intranasally (i.n.), and intravenously (i.v.) in mice. Intravenous administration resulted in the highest content of geniposide in plasma and brain, as it bypassed the absorption process. The C_max_ of i.v. administration was twice that of i.n. and 20 times that of i.g., while the T_max_ for intranasal and intragastric administration was 1 min and 30 min, respectively. The AUC of geniposide in the brain was lowest for i.g., then i.n., and highest for i.v. The bioavailability of geniposide via the nasal route was 85.38%, which is three times higher than gastric administration [151]. The results indicate that nasal and intravenous administration of geniposide allow for rapid and complete absorption, making them safe, convenient, inexpensive, and relatively efficient methods. Wang et al. found that the peak plasma concentration of geniposide was higher in solid lipid nanoparticles (SLNs) than in solution. Furthermore, in comparison to geniposide in solution, the mean residence time and t_1/2_ of geniposide-SLNs were significantly extended, leading to a remarkable 50-fold increase in relative bioavailability [152].

The coexisting ingredients during preparation can affect the pharmacokinetic parameters of geniposide [9]. Concurrently, administration can also alter the tissue distribution and pharmacokinetics of geniposide in mice [153, 154]. For instance, notoginsenoside R1 can enhance the gut absorption of geniposide by inhibiting intestinal motility, subsequently increasing its oral bioavailability [155]. Pan et al. observed an enhanced bioavailability of geniposide in middle cerebral artery occlusion model rats when combined with baicalin, possibly due to the inhibition of geniposide hydrolysis by baicalin. Conversely, combining geniposide with berberine resulted in decreased bioavailability, potentially attributed to berberine's promotion of geniposide hydrolysis [156].

#### Toxicology

The characterization of G. jasminoides in the Chinese Pharmacopoeia (Chinese Pharmacopoeia 2020) does not reference to its toxicity. However, several studies have indicated that geniposide, a crucial bioactive compound found in G. jasminoides, exhibits hepatotoxic and nephrotoxic effects, with hepatotoxicity being the primary safety concern [27]. High doses of G. jasminoides decoction have been shown to induce fatigue and lethargy in mice, along with hepatic impairment characterized by elevated AST and ALT activities and liver histopathological lesions [157]. Geniposide, administered at 300 mg/kg for three days in rats, can result in weight loss, diarrhea, weakness, and abnormal liver function tests. Furthermore, an increase in the relative liver weight and significant pathological changes were observed in the liver tissue [158]. Toxicological studies on rats have demonstrated that oral intake of geniposide at or above a dosage of 574 mg/kg can generate hepatotoxicity related to oxidative stress within 24–48 h, evidenced by elevated liver enzymes, increased liver weight, and pathological alterations. Affected rats exhibited immobility, lateral recumbency, reduced locomotion, faint respiration, blue feces, and diarrhea. In severe cases, mortality was observed. However, continuous administration for 90 days at dosages ranging from 24.3 to 72.9 mg/kg in rats did not result in evident hepatotoxicity [159]. In an extended-duration toxicity study, oral intake of geniposide at 100 mg/kg for a period of 26 weeks resulted in significant functional impairment in the kidney, liver, spleen, cerebrum, and thymus, accompanied by histopathological changes in these organs. Additionally, notable effects were observed in blood cell constituents as well as urinary bilirubin and urobilinogen levels [160]. The presentation of geniposide toxicity and side effects is detailed in Table 2.

**Table 2:** Toxicity and adverse effects of geniposide

Experimental type and model	Medicine and dose	Group	Administration	Duration	Toxicity and adverse effects	References
In vivo experiments in mice	G. jasminoides decoction: dosage unspecified	(1) Control: water; (2) Low-dose; (3) Medium-dose; (4) High-dose	Intragastric administration	7 days	Medium- and high-dose: Symptom: burnout, behavioral inactivit; Biochemical criterion: ALT↑, AST↑; Hepatic pathological alterations	[157]
In vivo experiments in male SD rats	GE: 100, 300 mg/kg	(1) Control: pure water; (2) Two GE-treated groups: 100, 300 mg/kg	Intragastric administration	3 days	300 mg/kg: Symptom: diarrhea, weakness, weight loss; Biochemical criterion: ALT↑, AST↑, ALP↑, γ-GT↑, TBA↑, t-CBAs↑; Liver weight↑; Hepatic pathological alterations	[158]
In vivo experiments in male and female SD rats	GE: 819.2, 1024, 1280, 1600, 2000, 2500 mg/kg	(1) Control: distilled water; (2) Six GE-treated groups: 819.2, 1024, 1280, 1600, 2000, 2500 mg/kg	Oral administration	14 days	Symptom: immobility, lateral recumbency, reduced locomotion, faint respiration, blue feces, diarrhea; Hepatic pathological alterations	[159]
	GE: 143.5, 287, 574, 861, 1148 mg/kg; Carbon tetrachloride: 2 mL/kg	(1) Control: distilled water; (2) Carbon tetrachloride; (3) Five GE-treated groups: 143.5, 287, 574, 861, 1148 mg/kg		24 h	GE ≥ 574 mg/kg: Biochemical criterion: ALT↑, AST↑, ALP↑, TB↑; Lliver weight↑	
	GE: 861 mg/kg	(1) Control: distilled water; (2) Four GE-treated groups: 12, 24, 48, 72 h		12, 24, 48, 72 h	Biochemical criterion: ALT↑, AST↑, ALP↑; Hepatic pathological alterations	
	GE: 24.3, 72.9 mg/kg;	(1) Control: distilled water; (2) Two GE-treated groups: 24.3, 72.9 mg/kg		90 days	No significant change	
In vivo experiments in male and female SD rats	GE: 25, 50, 100 mg/kg, 10 mL/kg	(1) Control: distilled water; (2) Three GE-treated groups: 25, 50, 100 mg/kg	intragastric administration	26 weeks	GE at 100 mg/kg: Symptom: diarrhea; green urine; polyuria; Biochemical criterion: BIL↑, URO↑; AST↑, K^+^↑; TBA↓, Crea↓; RBC↓, HGB↓, HCT↓, WBC↑; Organs weights: liver↑, spleen↑, kidney↑, brain↑, thymus↓; Hepatic and nephric pathological alterations	[160]

*GE* geniposide, ↓ decrease, ↑ increase, *ALT* Alanine transaminase, *AST* Aspartate aminotransferase, *SD* Sprague–Dawley, *ALP* Alkaline phosphatase, *γ-GT* γ-glutamytransferase, *TBAs* total bile acids, *t-CBAs* taurine-conjugated bile acids, *TB* Total bilirubin, *BIL* urobilirubin, *URO* urobilinogen, *Crea* Creatinine, *RBC* red blood cell, *HGB* hemoglobin, *HCT* hematocrit, *WBC* white blood cell

The method of administration significantly influences the hepatotoxicity of geniposide. Intranasal administration exhibits lower hepatotoxicity compared to intramuscular, intragastric, and intravenous routes [27]. Moreover, oral administration results in higher toxicity levels than intravenous administration [7]. Geniposide can be hydrolyzed to genipin in the intestinal tract by the enzyme β-D-glycosidase after oral intake [11]. In experiments with HepG2 cells treated with genipin and geniposide for 24 h, only genipin exhibited cytotoxicity, indicated by a concentration-dependent reduction in cell viability and an increase in LDH secretion. This finding suggests that the metabolization of geniposide into genipin contributes to its toxicity [161]. Additionally, genipin has demonstrated potential developmental toxicity to zebrafish embryos/larvae [162]. Further research has revealed that the synthesis of a dialdehyde intermediate upon hydrolysis is primarily responsible for hepatotoxicity induced by geniposide, which is affected by the intestinal pH value [163].

By employing the dose conversion methods between humans and laboratory animals, the pharmacologically active dose of geniposide used in rats at 100 mg/kg is converted to an equivalent dose of 1.6 mg/kg for humans [164], translating to a dose of 96 mg for a 60 kg adult. The recommended daily dosage of G. jasminoides fructus in clinical therapy for humans, as specified by the Chinese Pharmacopoeia 2020, is 6–10 g. This dosage range corresponds to a geniposide content of 108–180 mg (1.8%). Wu et al. have demonstrated that commercial G. jasminoides fructus typically contains a geniposide content ranging from 3.6% to 4.1% [28], suggesting that the geniposide dosage in clinical application may exceed the recommended geniposide content specified in the Chinese Pharmacopoeia 2020. To mitigate potential safety concerns, it is advisable to establish an upper limit for the standard content of geniposide in G. jasminoides fructus.

In summary, the toxicity of geniposide is contingent upon factors such as dosage, administration method, timing, and gut pH. In clinical practice, geniposide should be administered gradually in small dosages, with careful control of the duration of medication, preferably not exceeding 6 months. Currently, research on the toxic effects of geniposide, the primary extract from the fruit of the Chinese medicinal herb Zhizi, remains limited to animal and cellular experiments, with a lack of pertinent human studies. Nevertheless, Zhizi is widely used in clinical practice in Chinese medicine and is renowned for its excellent safety profile and efficacy. Although some patients may experience mild diarrhea after consuming large doses, there have been no clinical reports of liver function impairment caused by Zhizi. Therefore, ensuring the therapeutic and preventive effects of geniposide without inducing toxicities remains a significant focus for future research endeavors.

### Pharmacotherapeutic agents associated of geniposide

Geniposide, the main bioactive compound from G. jasminoides, has garnered extensive utilization in contemporary Chinese medicine preparation, including but not limited to Qing-kai-ling injection, Xing-nao-jing injection, Lian-zhi-fan solution, and Yin-zhi-huang injection or oral liquid [153, 165–167]. Additionally, novel formulations of geniposide have emerged in clinical practice, such as geniposide cubic liquid crystal gel and its ointment, and Shuluo powder injection (containing the active ingredients geniposide and paeoniflorin) [168, 169]. However, the use of geniposide as a therapeutic intervention for atherosclerotic cardiovascular disease remains infrequent in clinical practice.

In a clinical randomized controlled study on patients with coronary atherosclerotic heart disease and premature ventricular contractions, the modified formula of Zhizichi decoction combined with Danzhi Xiaoyao decoction demonstrated a significantly higher efficacy rate (92.31%) compared to bisoprolol treatment (75.56%). Concurrently, this study identified G. jasminoides as the principal bioactive substance in this herbal formulation, containing 15 potent components that target 180 action sites [170]. Additionally, an investigation into the plasma active ingredients in rats after oral administration of Zhizichi decoction has substantiated geniposide as its primary efficacious component [171]. The aforementioned findings indicate the clinical potential of geniposide in the treatment of atherosclerotic cardiovascular diseases, highlighting its potential as a novel therapeutic agent. Further investigation into the application value of geniposide in this domain is imperative.

## Discussion

Atherosclerotic cardiovascular disease (ASCVD) has become a major health threat worldwide, and its treatment strategy has long been a focus of discussion in the medical field. The treatment of ASCVD should be a comprehensive management process, including lifestyle adjustments, drug therapy, and surgical or interventional therapy when necessary. In the prevention and control of ASCVD, lifestyle intervention plays a pivotal role. Meanwhile, drug therapy must be precisely tailored to the individual differences of patients. With a deeper understanding of the pathogenesis of ASCVD, the development of new therapeutic targets and drugs has become increasingly urgent. At this juncture, the unique advantages of traditional Chinese medicine in the treatment of atherosclerosis are gradually being recognized. It intervenes in the pathological process of atherosclerosis through multiple targets and pathways, and geniposide, as a potential therapeutic drug, has opened up a new treatment pathway for ASCVD. Geniposide combats inflammation by regulating the release of inflammatory factors and mediators, obstructing monocytes adhesion to VECs, suppressing M1 macrophage polarization while enhancing M2 macrophage polarization, as well as attenuating the proliferation and migration of VSMCs. These mechanisms are associated with the regulation of miRNA-101 and miRNA-21 expression, along with the modulation of signaling pathways involving p38 MAPK, JNK, AKT, ERK, NF-κB, and TLR. Moreover, geniposide modulates immune cells and the reverse cholesterol transport, thereby limiting the formation of macrophage foam cells and improving lipid metabolism. Geniposide also inhibits PLA2 activity and TXA2 production, while simultaneously upregulating NO expression to suppress WPB exocytosis, thereby reducing the secretion of vWF and P-selectin. Consequently, this mechanism ameliorates platelet aggregation and thrombosis formation induced by endothelial injury. The protective effects of geniposide against mitochondrial dysfunction involve reducing oxidative stress products and inhibiting apoptosis in cardiomyocytes and VECs through modulation of the Bax/Bcl2/caspase-3 axis. Geniposide can regulate autophagy in macrophages, cardiomyocytes, and VECs by modulating key autophagy proteins and signaling pathways, including AKT, mTOR, TREM2, and Sirt3.

Furthermore, geniposide exerts a suppressive effect on oxidative stress damage and promotes the antioxidant system by attenuating NOX2 expression and enhancing antioxidant activities through the activation of Nrf2/HO-1, Sirt-1, and miRNA‑21/PTEN signaling pathways. Geniposide is not a primary hepatotoxic agent, but it can be hydrolyzed by β-D-glycosidase enzyme in the gut to produce hepatotoxic metabolites. The oral or intragastric administration of geniposide can induce toxicity through gastrointestinal circulation. Pharmacokinetic studies have demonstrated that the intranasal and intravenous administration of geniposide leads to rapid absorption and higher bioavailability compared to oral or intragastric administration. Therefore, the clinical dosage forms of geniposide therapeutic agents are mainly non-oral preparations, including Qing-kai-ling injection, Yin-zhi-huang injection, geniposide cubic liquid crystal gel and its ointment, and Shuluo powder injection [165, 167–169]. The significant role of geniposide in combating atherosclerosis cannot be overlooked, given its considerable clinical application potential. However, it is undeniable that current research on the use of geniposide in atherosclerosis faces certain limitations, necessitating further studies to validate and refine these findings. Below are the limitations of current studies and potential directions for future research:

1. *Inadequate mechanistic exploration* (a) Macrophage polarization: While geniposide has been shown to modulate macrophage polarization through regulating expressions like CXCL14, ultimately improving atherosclerosis, a deeper understanding of the underlying molecular mechanisms and signaling pathways remains crucial. (b) Autophagy and apoptosis in VSMCs: The association between autophagy and apoptosis of VSMCs with atherosclerosis is well-established [172–174], but the role of geniposide in this context remains unexplored. While previous studies have demonstrated geniposide can activate autophagy to improve apoptosis in osteoblasts and kidney podocytes [118, 119], it is unclear whether it can modulate the interplay between apoptosis and autophagy in the cardiovascular system. This presents a novel avenue for investigating the potential therapeutic role of geniposide in atherosclerosis. (c) Gut microbiota: Geniposide can regulate gut flora and fortify the gut barrier to alleviate inflammation. However, it remains uncertain whether geniposide can ameliorate atherosclerotic damage by regulating gut microbiota. Therefore, further experimental validation is warranted to confirm the hypothesis that geniposide may mitigate the risk of ASCVD through modulation of intestinal flora-related factors expression, downregulation of TMAO levels, and upregulation of SCFAs levels.
2. *Inadequate assessment of side effects* Most studies on geniposide toxicity primarily focus on oral or gastric administration, with limited research conducted on alternative routes of administration. It is still unknown whether geniposide administered nasally or intravenously injection will cause toxicity. Therefore, further experiments are needed to conduct a more comprehensive assessment of the side effects of geniposide under different administration methods.
3. *Insufficient clinical research data* (a) Current pharmacological, pharmacokinetic, and toxicological studies on geniposide have mainly been conducted in animal and cellular models, which, despite yielding positive results, differ physiological and pathological differences from humans. Long-term follow-up studies are necessary to evaluate the safety and potential side effects of geniposide in prolonged use. (b) Advanced pharmacokinetic methods like mass spectrometry should be utilized to study the absorption, distribution, metabolism, and excretion of geniposide in humans. (c) Its efficacy should be assessed through pharmacodynamic indicators such as biomarkers and clinical symptom improvement, and its mechanism of action explored. (d) Beyond traditional toxicity assessment, attention should be paid to geniposide's long-term effects on specific organs like the liver and kidneys, using advanced imaging techniques and biomarker monitoring.

In conclusion, geniposide has exhibited promising potential in the fight against atherosclerosis. However, further research and exploration are required to fully validate and optimize its clinical application. Future studies should delve deeper into its molecular mechanisms and signaling pathways, conduct rigorous assessments of drug interactions and potential side effects, and explore innovative drug delivery methods and formulations. Through these endeavors, we anticipate acquiring more robust evidence proving the potential of geniposide as a therapeutic agent for atherosclerosis, ultimately offering patients a more effective and safer treatment option.

## Data Availability

Not applicable.

## Abbreviations

IL-S: Interleukins
TLR4: Toll-like receptor 4
PI3K: Phosphoinositide 3-kinase
VSMC: Vascular smooth muscle cell
vWF: Von Willebrand factor
PLA2: Phospholipase A2
TXA2: Thromboxane A2
AMPK: Adenosine monophosphate-activated protein kinase
TREM2: Trigger receptor expressed on myeloid cells 2
PAI-1: Plasminogen activator inhibitor-1
AKT: Protein kinase B
mTOR: Mechanistic target of rapamycin
iNOS: Inducible nitric oxide synthase
Arg-1: Arginase-1
NR4A1: Nuclear receptor subfamily 4 group A member 1
Sirt1: Sirtuin1
JNK1/2-c: Jun N-terminal kinase
GLP-1R: Glucagon-like peptide-1 receptor
CD: Cluster of differentiation
ROS: Reactive oxygen species
MAPK: Mitogen-activated protein kinase
GSH-PX: Glutathione peroxidase
DKK1: Dickkopf-related protein-1
TXNIP: Thioredoxin-interacting protein
MKP-1: Mitogen-activated protein kinase (MAPK) phosphatase-1
PTEN: Phosphatase and tensin homolog
Nrf2: Nuclear factor erythroid 2-related factor
PKC: Protein kinase C
VCAM: 1-Vascular cell adhesion molecule-1
CAT: Catalase
MMPs: Matrix metalloproteases
CYP7A1: Cholesterol 7 alpha-hydroxylase
I-BABP: Ileal-bile acid-binding protein
ASBT: Apical sodium-dependent bile acid transporter
OST: Organic solute transporte
FXR: Farnesoid X receptor
SR-A/B: Scavenger receptor A/B
ABCA1: Adenosine triphosphate-binding cassette transporter A1
SOD: Superoxide dismutase
M1/2: Macrophage1/2
eNOS: Endothelial nitric oxide synthase
FOXP3: Forkhead box protein P3
WPB: Weibel-Palade bodies
AA: Arachidonic Acid
p62: Substrate ubiquitin binding protein of autophagy
ATG: Autophagy protein
TGF-β1: Transforming growth factor-β1
MDA: Malondialdehyde
VEC: Vascular endothelial cell
ALT: Alanine transaminase
AST: Aspartate aminotransferase
SD: Sprague–Dawley
ALP: Alkaline phosphatase
γ-GT-γ: Glutamytransferase
TBAs: Total bile acids
t-CBAs: Taurine-conjugated bile acids
TB: Total bilirubin
BIL: Urobilirubin
URO: Urobilinogen
NS: Normal saline
PA: Palmitic acid
LAD: Left anterior descending
TTC: Triphenyltetrazole chloride
NRVMs: Neonatal rat ventricular myocytes
